# Optimal treatment for small HCC (<3 cm): Resection, liver transplantation, or locoregional therapy?

**DOI:** 10.1016/j.jhepr.2023.100781

**Published:** 2023-04-25

**Authors:** Xiao Wu, Ryan Peter Lokken, Neil Mehta

**Affiliations:** 1 Department of Radiology and Biomedical Imaging, University of California, San Francisco, CA, USA; 2 Department of General Hepatology and Liver Transplantation, University of California, San Francisco, CA, USA

**Keywords:** Liver transplantation, Locoreginoal therapy, Resection, Small hepatocellular carcinoma

## Abstract

Hepatocellular carcinoma (HCC) remains the most common form of liver cancer, accounting for 90% of all primary liver cancers. Up to 30% of HCC cases could be small (2–3 cm in diameter) at the time of diagnosis with advances in imaging techniques and surveillance programmes. Treating patients with early-stage HCC can be complex and often requires interdisciplinary care, owing to the wide and increasing variety of treatment options, which include liver resection, liver transplantation, and various locoregional therapies offered by interventional radiology and radiation oncology. Decisions regarding the optimal management strategy for a patient involve many considerations, including patient- and tumour-specific characteristics, as well as socioeconomic factors. In this review, we aim to comprehensively summarise the commonly used therapies for single, small HCC (<3 cm), with a focus on the impact of tumour size (<2 cm *vs*. 2-3 cm), as well as a brief discussion on the cost-effectiveness of the different treatment options.

**Key points**

• Treating patients with early-stage hepatocellular carcinoma (HCC) can be complex, owing to the availability of many different treatment options, including liver resection, liver transplantation (LT), and various locoregional therapies.
• For patients with single, small HCC (<3 cm) without macrovascular invasion or extrahepatic disease, treatment approaches should be differentiated based on whether the tumour exceeds 2 cm.
• Outcomes after liver resection are favourable for single, small HCC, especially those <2 cm, with high overall survival and low recurrence rates.
• While primary LT has been recognised as the best curative treatment for HCC with underlying cirrhosis, it may not confer significant additional survival benefit compared to alternative treatments for HCC <2 cm. For patients with HCC measuring 2–3 cm, bridging to LT with locoregional therapy is recommended.
• Thermal ablation is recognised as an alternative treatment strategy for HCC that is not amenable to resection or transplant, with similar survival outcomes to resection reported in the literature.
• Radiation segmentectomy has shown promise as a potentially curative therapy for single, small HCC in recent trials and prospective studies.
• Additional locoregional therapies include transarterial chemoembolisation (TACE), TACE followed by ablation, and external beam radiation. Given the multiple available treatment options, the complexity of HCC care, and the high economic and societal burden of HCC, decision analyses can be helpful in deciding the optimal treatment approach.

## Introduction

Hepatocellular carcinoma (HCC) remains the most common form of liver cancer, accounting for 90% of all primary liver cancers.^1^ The burden of HCC continues to rise, with a projected incidence of over 1 million cases by 2025, partly owing to the increasing prevalence of non-alcoholic steatohepatitis (paralleling increases in obesity and metabolic syndrome), which is becoming the fastest growing cause of HCC in the West.^2^ Besides being one of the leading causes of cancer mortality, HCC also has significant financial implications, causing direct healthcare costs of >$400 million and productivity losses of $50 million in the US annually.^3^^,^^4^

At the time of HCC diagnosis, it has been estimated that 10-15% of HCC cases are small tumours (<2–3 cm) in the West compared to 30% in Japan, and this proportion may be even higher with the wider implementation of routine surveillance programmes in high-risk patients.[5], [6], [7], [8], [9] Challenges of managing early-stage HCC not only arise from patient-specific factors, such as baseline liver function and co-morbidities, but also from the increasing number of treatment options as more evidence emerges.

In this review, we will summarise the commonly used therapies for single, small HCC (defined as <3 cm) in patients without macrovascular invasion or extrahepatic disease, with a focus on the impact of tumour size (<2 cm or T1 HCC *vs.* 2–3 cm or T2 HCC).

## Liver resection

Surgical resection has been recommended as a first-line treatment for suitable candidates with very early (Barcelona Clinic Liver Cancer [BCLC] stage 0) and early (BCLC A) stage HCC with a single lesion.[10], [11], [12] In the 2022 update of the BCLC prognosis and treatment strategy, resection was deemed appropriate for patients with BCLC 0 disease who are liver transplant (LT) candidates and those with BCLC A disease and a single HCC lesion.^13^ The definition of surgical candidacy can be highly dependent on institutional and individual surgeon experiences, but common key considerations include remnant liver function, absence of clinically significant portal hypertension, lesion location and patient’s performance status.^14^^,^^15^

Overall survival (OS) after liver resection has been reported to be up to 50–70% at 5-year follow-up for patients with small HCC and preserved liver function, with more favourable outcomes for patients with tumours smaller than 2 cm.[16], [17], [18], [19], [20] Zhou *et al.* included 1,000 patients with small HCC (<5 cm) who underwent liver resection, with complete resection achieved in 805 of them. The 5-year survival rate was 82.5% for patients with tumours smaller than 2 cm, and 66.3% in patients with a tumour of between 2–3 cm.^17^ Seshadri *et al.* analysed the National Cancer Database from 1998 to 2011. The median OS was 57 months in all AJCC (American Joint Committee on Cancer) clinical stage I patients, and 88 months for those with a HCC smaller than 2 cm.^21^ The risk of tumour recurrence can be high, with a reported disease-free survival rate of 30–40% at 5 years, which is even lower at 28% in patients with cirrhosis.^16^^,^^20^ Pinna *et al.* showed plateauing of the Kaplan Meier curve for both disease-free survival (DFS) and OS after 10-14 years, with “statistical cure” in 24.1–40.5% of patients (using DFS and OS curves, respectively).^22^ In patients with small HCC after resection, recurrences were more likely to be within Milan criteria. Among all the recurrences after resection of tumours within Milan criteria, most recurrences were still within Milan criteria (58–62%).^23^^,^^24^ A high proportion of these patients were eligible for salvage transplant, which appears to be associated with similar outcomes to primary LT for HCC.^16^^,^^25^^,^^26^

Increasing experience with minimally invasive surgery (MIS, including laparoscopic and robotic-assisted liver resection) means that more patients may now be considered surgical candidates – including patients with tumours in unfavourable locations (such as posterior superior segments I, IVa, VII and VIII) or those with portal hypertension – without compromising long-term outcomes.^27^ Better short-term outcomes have been reported with MIS, including decreased blood loss, transfusion requirements, % of positive resection margins and length of hospital stay.^28^ No statistically significant difference was found between MIS and open resection in terms of long-term survival on propensity matched analysis. Given its minimally invasive nature, MIS has also been compared with thermal ablation (percutaneous or laparoscopic) and multiple studies have shown comparable OS but higher DFS with MIS.^29^^,^^30^ A meta-analysis by Jin *et al*. pooled data from seven randomised-controlled trials (RCTs) comparing laparoscopic hepatectomy and radiofrequency ablation (RFA). The authors found that laparoscopic hepatectomy was associated with lower recurrence but higher immediate complication risks.^31^

A summary of the literature is presented in Table 1.

**Table 1:** Summary of studies on liver resection.

Study, year	Patient population	Cohort size	Resection technique	OS			DFS		
				3-year	5-year	10-year	3-year	5-year	10-year
Giuliante *et al.*, 2012^20^	HCC <3 cm	588	Not specified		52.8%	20.3%		32.4%	21.7%
Lee *et al.*, 2021^30^	HCC <3 cm	251	Laparoscopic	97.9%			74.4%		
Pinna *et al.*, 2018^22^	Patients who underwent resection, 38.5% beyond T2	2,068	Not specified	70.5%	57.3%	33.9%	45.3%	33.6%	19.2%
Poon *et al.*, 2002^16^	HCC within Milan criteria	473	Open	76%	70%	35%	50%	36%	22%
Thelen *et al.*, 2013^18^	Patients who underwent resection, median HCC size 7 cm	110	Open	66%	50%		53%	42%	
Zhou *et al.*, 2001^17^	HCC <5 cm	1,000	Open		62.7%	46.3%			

DFS, disease-free survival; HCC, hepatocellular carcinoma; OS, overall survival.

## Liver transplantation

Primary LT has long been recognised as the best curative treatment for patients with HCC and underlying cirrhosis, as acknowledged by all national and international guidelines.^10^ The selection of transplant candidates is especially critical due to worldwide organ shortages, necessitating careful consideration and balance of i) post-transplant outcomes, ii) waitlist dropout risks and iii) alternative curative treatment options.

### Very early-stage HCC (<2 cm)

In the US, as opposed to patients meeting T2 criteria, T1 patients have not been eligible for priority listing since 2004.^32^ The rationale behind the allocation policy change includes i) much lower dropout risk among patients with T1 stage disease, and ii) a relatively high false positive rate on the explant (as high as 31%). The risk of disease progression in T1 patients was reported to be low, at 8.7% when listed from 2019 to 2021, especially with adequate bridging therapy.^33^^,^^34^ The second factor is due to the existing diagnostic challenge associated with very small liver lesions which do not always exhibit the characteristic arterial hyperenhancement and delayed washout on quadruple phased imaging. This is underscored by the LI-RADS (Liver Imaging Reporting and Data System), widely used for the standardised reporting of HCC screening, which specifically used 2 cm as a size cut-off below which a lesion must exhibit major features in addition to arterial hyperenhancement before it can be graded as LR-4 or 5 (probably and definitely HCC).^35^

While LT may not confer a significant survival benefit for patients with T1 HCC and well compensated cirrhosis, its urgency increases in patients with decompensated cirrhosis. Mehta *et al.* have proposed a “wait and not ablate” approach, consisting of imaging surveillance with contrast-enhanced CT or MRI every 3 months without immediate intervention. In the US, if the tumour progresses to within T2 HCC, the patient can then receive priority listing, based on model for end-stage liver disease (MELD) exception points, to facilitate LT. Among 114 patients under a “wait and not ablate” strategy, only 9.0% progressed directly from stage T1 HCC to beyond T2 at 24 months.^36^ However, no large prospective studies or RCTs have been performed to validate this strategy.

### Early-stage HCC (2–3 cm)

LT in patients with a single HCC lesion of size 2–3 cm is associated with excellent long-term outcomes. With a median follow-up of 8.4 years (IQR 3.7–12.9), the OS plateaued at 71.6% and the DFS was 75.0% at 10-year follow-up among patients with HCC <3 cm after LT in a study by Ivanics *et al.*^37^ Multiple studies have shown lower mortality and recurrence risks of patients undergoing transplant compared to resection at 5 years with comparable complication risks.^38^^,^^39^ However, small retrospective cohort studies have suggested similar outcomes, based on national averages, for patients who undergo LT and patients with early-stage HCC and Child-Pugh A cirrhosis who were denied LT for non-tumour reasons.^40^

To prevent progression of disease while on the transplant waitlist, it is recommended that patients receive locoregional bridging therapies.^10^ However, no specific modality has been recommended among options including percutaneous thermal ablation, transarterial chemoembolisation (TACE), transarterial radioembolisation (TARE), and possibly stereotactic beam radiation therapy (SBRT). Few prospective studies have been performed to directly compare the different bridging modalities for single, small HCC, and clinical practice has been constantly evolving with increased experiences with the various options. Currently, TACE remains the most used modality internationally, followed by ablation and TARE.^41^^,^^42^ In a retrospective analysis, Sapisochin *et al.* compared SBRT, TACE, and RFA as bridging therapies, and found comparable dropout rates among the three groups. In explant analysis after LT, RFA was associated with the highest level of tumour necrosis, with post-transplant OS being similar.^43^ Doyle *et al.* identified tumour size greater than 2 cm and alpha-fetoprotein level at the time of ablation between 100 and 1,000 ng/ml as predictors of post-RFA recurrence beyond Milan criteria.^44^

Additional considerations while choose one bridging modality over another should also incorporate cost-effectiveness analyses. Among the various interventional radiology options, under most circumstances, ablation has the lowest procedural cost and TARE the highest, as it is usually a two-part procedure.^45^ Post-procedural complications, disease progression and need for additional locoregional therapy can have greater financial implications than the initial bridging therapy itself. Effectiveness, commonly measured by QALYs (quality-adjusted life years) which account for both quality and length of life, are affected by a similar set of factors. A recent cost-effectiveness analysis comparing different bridging modalities based on United Network for Organ Sharing data found ablation to be the most cost-effective strategy when compared to TACE and TARE, primarily because it was associated with the lowest dropout risk.^45^

### LT survival benefit for single, small HCC

While transplant may offer the longest OS and DFS in patients with single, small HCC (<3 cm), the benefit must be weighed against the implications to other patients on the transplant waitlist. It has been proposed that there might be decreased urgency in listing patients with lower risk HCC and otherwise well compensated liver disease.^34^ The concept of “transplant benefit” has been proposed, which is defined as number of years gained by LT subtracted from the number of years offered by alternative treatments.^46^ For example, immediate hepatic resection of solitary HCC in patients without portal hypertension may even be associated with higher OS in regions with long wait times, as it reduces the risk of disease progression or mortality on the waitlist.^38^ The true urgency of LT in resectable HCC with well-preserved function has been discussed in several studies. In patients with good radiologic response to locoregional therapy and a low likelihood of liver disease progression (*e.g*. following alcohol cessation or effective antiviral treatment), the urgency of LT has been deemed very low.^47^ In addition to small tumour size, predictors of low dropout risk include MELD <15, Child-Pugh A, and alpha-fetoprotein <20 ng/ml.^48^

## Locoregional therapies

### Thermal ablation

Thermal ablation has long been considered the leading alternative treatment strategy for small HCCs at locations that are not amenable to resection and/or in patients with portal hypertension who are not transplant candidates. RFA is the best studied mechanism, with several RCTs comparing it with resection but showing mixed results. RCTs were performed by Fang *et al.* (lesions <3 cm with most patients having only a solitary lesion) and Chen *et al.* (solitary lesions <5 cm).^49^^,^^50^ They found no statistically significant difference in terms of OS and DFS at 3- and 4-year follow-up respectively, with lower complication rates and shorter hospital stays in the RFA group. Meanwhile, in an RCT by Huang *et al.*, resection was associated with a higher 5-year OS rate in the subgroup of patients with solitary <3 cm HCC (82.2% for hepatic resection *vs.* 61.4% for RFA).^51^ A meta-analysis of five RCTs also showed RFA to be associated with lower 5-year OS and higher recurrence, although trial sequential analysis showed more trials would be needed to control for random errors.^52^

Cost-effectiveness analyses have been performed comparing RFA and hepatic resection. Cucchetti *et al.* concluded that RFA was the more cost-effective strategy for patients with very early HCC (single, <2 cm) and 2–3 nodules <3 cm, while resection was the better strategy for single 3-5 cm HCCs.^53^ Ikeda *et al.* found RFA to be superior to surgery for HCC <3 cm from the Japanese perspective.^54^ Huang *et al.* also reported that RFA resulted in improved quality of life compared to resection.^55^

Both RFA and microwave ablation (MWA) rely on heat-based thermal toxicity to cause coagulative necrosis, but MWA has a more predictable ablation zone due to avoidance of the heat-sink effect observed with RFA.^56^ The difference is more relevant for tumours close to vessels, or larger tumours. Among the published studies, comparable outcomes have been reported for small HCCs.^57^ Generally, a major limitation of both RFA and MWA compared to surgical resection has been the possibility of leaving an incomplete ablative margin due to the lack of direct intra-operative visualisation. Recent advances, such as the use of contrast-enhanced ultrasound-CT/MR image fusion, have been shown to improve the assessment of the ablative margin intra-operatively, leading to lower local recurrence rates.^58^

### Transarterial radioembolisation/radiation segmentectomy

TARE was initially applied in HCC as an alternative to TACE for patients with portal vein thrombosis.^59^ The initially technique often involved administration of radioactive microspheres into an entire lobe, and its efficacy was primarily limited to patients with intermediate to advanced HCCs in the 2000s to early 2010s.[60], [61], [62] Subsequent evolution of the procedure shifted the paradigm to treatment of early-stage disease with radiation segmentectomy (RS), defined as an ablative dose (>200-400 Gy) delivered to one to two segmental artery/arteries.^63^

TARE was compared with TACE via a phase II RCT in 2016 in the PREMIER trial consisting of a total of 45 patients with BCLC A or B HCC. The study showed significantly longer time-to-progression in the TARE group (>26 months *vs*. 6.8 months), which is sustained in larger observational studies.^64^^,^^65^ Several studies published in 2021 showed higher radiation dose to be associated with higher response rates. The multicentre LEGACY study by Salem *et al.* included 162 patients with a median tumour size of 2.7 cm undergoing TARE as neoadjuvant therapy for resection or transplantation. The 3-year OS rates were 86.6% and 92.8% after resection and LT, respectively. The local progression rate was 0 at 24 months, with a PFS of 93.9%. An explant analysis of 45 patients suggested 400 Gy as a threshold dose to achieve complete pathologic necrosis.^66^ Based on the LEGACY results, the most recent BCLC guidelines now suggest TARE as well as TACE as alternatives when ablation is not feasible.^13^

In 2022, the RASER trial (single-centre, single-arm) investigated RS as a curative treatment for unresectable, single HCC <3 cm. Within a median follow-up of 691 days, median time to target lesion progression was not reached. The risk of target lesion progression was 4% and 12% at 1- and 2-year follow-up, respectively. Eight patients received an LT eventually and the target lesion showed complete necrosis in the explants.^67^ Previous retrospective studies have also shown similar results, with reported median OS of 53–59 months for single HCC <4–5 cm, with longer target lesion PFS compared to MWA.^68^^,^^69^ A cost-effectiveness analysis from the UK perspective compared TARE to bland transarterial embolisation and TACE (both conventional and drug-eluting beads) in patients with early to intermediate stage HCC and concluded TARE to be cost-saving.^70^

### Transarterial chemoembolisation followed by ablation

A common combination therapy used with curative intent for HCC is TACE followed by RFA or MWA (TACE-ablation) within 0–4 weeks. A meta-analysis by Ni *et al.* pooled eight RCTs comparing TACE-ablation and RFA alone, which showed TACE-ablation was associated with similar OS for tumours <3 cm at 1-, and 3-year follow-up. The combination therapy only showed survival benefits for larger tumours.^71^ A retrospective propensity score-matched study comparing RS with TACE-ablation for unresectable, single HCC <3 cm, showed similar time-to-progression and OS between the two groups.^72^ No survival difference was found between TACE-RFA and TACE-MWA but there are studies suggesting better response rates with the latter.^73^^,^^74^

### Transarterial chemoembolisation

Although not commonly used as a curative monotherapy, TACE remains the most commonly used bridging therapy in patients with early-stage HCC awaiting LT.^33^ Often studied as primary treatment option for intermediate stage HCC, TACE has also been shown to reduce waitlist dropout as a bridging therapy to LT.^33^ Burrel *et al.* examined the efficacy of TACE with drug-eluting beads (DEB-TACE) in 41 patients with BCLC A HCC, reaching a median OS of 54.2 months.^75^ Some operators may prefer TACE over ablation, particularly in potential transplant candidates, due to the rare complication of tumour seeding along the ablation tract.^76^ Response to pre-transplant TACE has been shown to be a valuable predictor of post-transplant outcome, with lower risk of tumour recurrence and possible longer OS (did not reach statistical significance) in patients who did not progress after pre-transplant TACE compared to those who did.^77^^,^^78^ DEB-TACE and conventional lipiodol-based TACE had similar efficacy in terms of response rates and OS, but DEB-TACE was associated with fewer peri-procedural adverse events.^79^^,^^80^ A phase II RCT was performed comparing TACE and TARE for 17 and 18 patients within Milan criteria, respectively. The study showed significantly longer time-to-progression with TARE (not reached) compared to TACE (6.8 months), although the study was terminated early due to slow accrual.^64^

### External beam radiation

External beam radiation therapy (EBRT), including proton beam therapy (PBT) and SBRT, can be considered as an alternative to percutaneous thermal ablation. Multiple retrospective and prospective studies have been performed to investigate the efficacy of EBRT as a curative treatment for small HCCs, demonstrating similar outcomes to those achieved with RFA and MWA.[81], [82], [83] Matthew *et al.* conducted a long-term retrospective follow-up of 297 patients after SBRT, and found SBRT to be associated with higher OS and local control for HCC <3 cm.^84^ SBRT has also been shown to be an effective bridging therapy to LT in small retrospective studies, with a median time-to-progression of 10-14 months prior to LT.^85^^,^^86^ No RCT was available on SBRT as a bridging therapy to LT.^87^ A phase III RCT performed in Korea in 2021 compared PBT with RFA for one or two residual or recurrent HCC(s) <3 cm and concluded non-inferiority of PBT at 4-year follow-up in terms of PFS and OS.^88^ EBRT can be associated with radiation-induced liver injury and can cause ulceration in the stomach or bowel, so EBRT candidates must be selected carefully.^89^ A prospective longitudinal study also showed minimal impact of SBRT on overall quality of life, with only temporarily worsened appetite and fatigue.^90^

A summary of the literature is presented in Table 2.

**Table 2:** Summary of studies on locoregional therapies.

Ablation		TACE		TARE		TACE-ablate		SBRT	
Study, year, design	Main findings	Study, year, design	Main findings	Study, year, design	Main findings	Study, year, design	Main findings	Study, year, design	Main findings
Chen *et al.*, 2006,^50^ RCT comparing LR and RFA for HCC <5 cm	Similar OS and DFS between LR (n = 90) and RFA (n = 71). Lower complications with RFA.	Burrel et a., 2012,^75^ DEB-TACE in BCLC A patients	Median OS 54.2 months (n = 104)	LEGACY study, 2021,^66^ multicentre, single-arm, retrospective study of TARE for solitary HCC <8 cm	3-year OS 86.6% and 92.8% after TARE (n = 162) with LR and LT. 400 Gy as a threshold dose for CPN	Biederman *et al.*, 2017,^72^ RS *vs.* TACE-ablate in HCC <3 cm	Similar TTP 11.6 in TACE-ablate (n = 80) and 11. 1 RS (n = 41) groups, after propensity matching	Moore *et al.*, 2017,^86^ retrospective study on SBRT as bridge to LT	Median progression free survival (local or distant) was 14.0 months for non-transplanted patients (n = 23)
Huang *et al.*, 2010,^51^ RCT comparing LR and RFA for HCC within Milan criteria	LR (n = 122) was associated with higher OS and lower recurrence compared to RFA (n = 115)	Salem et a., 2016,^64^ Phase II trial on cTACE and TARE in BCLC A and B patients	TARE (n = 24) provides significantly longer TTP (18.6 months *vs.* 17.7 months) compared to cTACE (n = 21)	TRACE, 2022 (92), Phase II trial, RS *vs.* DEB-TACE in BCLC A and B patients	MTP 17.1 months in RS (n = 38), 9.5 months in DEB-TACE (n = 34), study terminated early due to efficacy of RS	Abdelaziz *et al.*, 2017,^74^ retrospective comparing TACE-MWA and TACE-RFA in HCC <5 cm	TACE-MWA (n = 45) lead to better response rate compared to TACE-RFA (n = 22), no difference in OS	Matthew *et al.*, 2020,^84^ multicentre retrospective study	Higher OS and local control for HCC <3 cm (n = 296)
Fang *et al.*, 2013,^49^ RCT comparing RFA and LR for HCC <3 cm	Similar local control and OS between LR (n = 60) and RFA (n = 60). Shorter hospital stay with RFA			RASER trial, 2022,^67^ single centre, single-arm on RS in HCC <3 cm	Risk of target lesion progression 4% and 12% at 1- and 2-year (n = 29). Time to target progression > 23 months				

BCLC, Barcelona Clinic Liver Cancer; CPN, compete pathologic necrosis; cTACE, conventional TACE; DEB-TACE, drug-eluting bead TACE; DFS, disease-free survival; HCC, hepatocellular carcinoma; LR, liver resection; LT, liver transplantation; MTP, median time to progression; MWA, microwave ablation; OS, overall survival; RCT, randomised-controlled trial; RFA, radiofrequency ablation; RS, radiation segmentectomy; SBRT, stereotactic beam radiation therapy; TACE, transarterial chemoembolisation; TARE, transarterial radioembolisation; TTP, time-to-progression.

## Conclusions

**Fig. 1:**
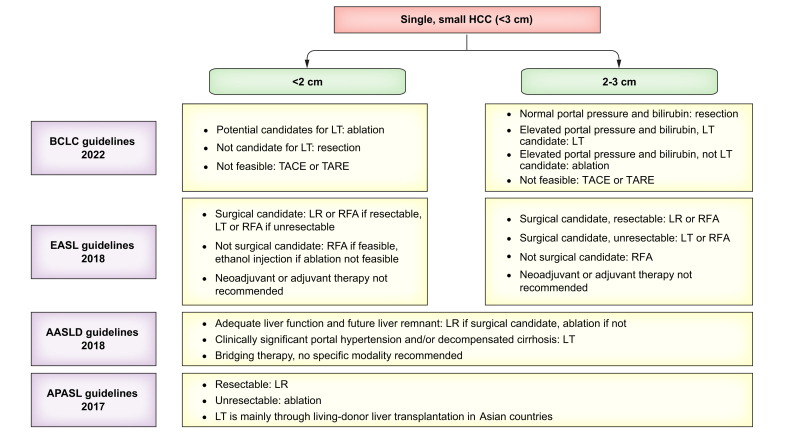
A summary of current national and international guidelines on the treatment of single, small HCC (<3 cm). AASLD, American Association for the Study of Liver Diseases; APASL, Asia Pacific Association for the Study of the Liver; BCLC, Barcelona Clinic Liver Cancer; EASL, European Association for the Study of the Liver; HCC, hepatocellular carcinoma; LR, liver resection; LT, liver transplantation; RFA, radiofrequency ablation; TACE, transarterial chemoembolisation; TARE, transarterial radioembolisation.

Treatment for patients with single HCC <3 cm can be complex, especially given the multiple available strategies reviewed above, and emerging modalities with proven safety and efficacy. Even though several RCTs have shown the promise of minimally invasive curative treatments, such as RS, TACE-ablate, and EBRT, larger prospective studies with longitudinal follow-up are needed to assess their longer-term efficacies more robustly. A summary of current national and international guidelines is presented in Fig. 1.

The decision to choose one strategy over another involves many factors, and would require thorough comparison with other available options, patient- and tumour-specific characteristics, as well as socioeconomic considerations. Given the relatively high medical costs associated with HCC and possibly underlying liver disease, and its long-term impact on a patient’s daily life, it is important to integrate decision-analysis, which can synthesise the available evidence from difference aspects, into patient care. A systematic review by Yuen *et al.* found 27 cost-effectiveness analyses on interventions for adults with HCC. Among them, seven studies included patients with early-stage HCC in the US.^91^ A summary of prior cost-effectiveness studies is provided in Table 3.

**Table 3:** A summary of studies on cost-effectiveness.

Study, year	Country	Strategy/strategies compared	Patient population	Conclusion
Cucchetti *et al.*, 2013^53^	Italy	LR *vs.* RFA	Early HCC	RFA is more cost-effective in T1 HCC; LR is more cost-effective in T2 HCC
Landman *et al.*, 2011^92^	US	Primary LT, LR + salvage LT *vs.* LRT + salvage LT	Within Milan criteria	Primary LT is more cost-effective compared to LR or LRT followed by salvage LT
Lim *et al.*, 2015^93^	US, Switzerland, Singapore	LR *vs.* LT	Within Milan criteria	LR is more cost-effective in all three countries
Llovet *et al.*, 2002^94^	Spain	LR, LRT or no treatment while awaiting for LT	Within Milan criteria	LR is cost-effective when waiting time >1 year, LRT is cost-effective for all waiting times
Maegawa *et al.*, 2019^95^	US	Primary LT *vs.* LR + salvage LT	Within Milan criteria	LT + salvage LT is more cost-effective
Manas *et al.*, 2021^70^	UK	TARE, cTACE, DEB-TACE, and TAE	Unresectable HCC that are candidates of transarterial therapy	TARE was cost-effective in early to intermediate HCC
Naugler and Sonnenberg, 2010^96^	US	TACE *vs.* RFA via immediate treatment or monitoring	Newly diagnosed HCC <2 cm in cirrhosis	Immediate treatment resulted in longer life expectancy, with RFA better than TACE
Rostambeigi *et al.*, 2014^97^	US	TACE *vs.* TARE	BCLC A-C patients	TARE was more cost-effective in BCLC-C disease, and borderline in BCLC-B disease
Sarasin *et al.*, 1998^98^	US	LR *vs.* LT	Resectable HCC <5 cm	LT is more cost-effective if waiting time does not exceed 6-10 months

BCLC, Barcelona Clinic Liver Cancer; cTACE, conventional TACE; DEB-TACE, drug-eluting bead TACE; HCC, hepatocellular carcinoma; LR, liver resection; LRT, locoregional therapy; LT, liver transplantation; RFA, radiofrequency ablation; TACE, transarterial chemoembolisation; TAE, transarterial embolisation; TARE, transarterial radioembolisation.

## Financial support

This study was not supported by any funding.

## Authors' contributions

XW: study conception and design, literature review, data collection, analysis and interpretation of results, draft manuscript preparation, manucript editing. RPL: study conception and design, literature review, manucript editing, supervision. NM: study conception and design, literature review, analysis and interpretation of results, manucript editing, supervision All authors reviewed and approved the final version of the manuscript.

## Conflict of interest

The authors declare no conflicts of interest that pertain to this work.

Please refer to the accompanying ICMJE disclosure forms for further details.
